# Identification of a novel lymphoid population in the murine epidermis

**DOI:** 10.1038/srep12554

**Published:** 2015-07-30

**Authors:** Francisca F. Almeida, Mari Tenno, Joanna Brzostek, Jackson LiangYao Li, Gabriele Allies, Guillaume Hoeffel, Peter See, Lai Guan Ng, Hans Jörg Fehling, Nicholas R. J. Gascoigne, Ichiro Taniuchi, Florent Ginhoux

**Affiliations:** 1 Singapore Immunology Network (SIgN), Agency for Science, Technology and Research (A*STAR), 138648, Singapore; 2 RIKEN Center for Integrative Medical Sciences (IMS), Japan; 3 Department of Microbiology, Yong Loo Lin School of Medicine, National University of Singapore, Singapore; 4 Institute of Immunology, University Clinics Ulm, Ulm, Germany

## Abstract

T cell progenitors are known to arise from the foetal liver in embryos and the bone marrow in adults; however different studies have shown that a pool of T cell progenitors may also exist in the periphery. Here, we identified a lymphoid population resembling peripheral T cell progenitors which transiently seed the epidermis during late embryogenesis in both wild-type and T cell-deficient mice. We named these cells ELCs (Epidermal Lymphoid Cells). ELCs expressed Thy1 and CD2, but lacked CD3 and TCRαβ/γδ at their surface, reminiscent of the phenotype of extra- or intra- thymic T cell progenitors. Similarly to Dendritic Epidermal T Cells (DETCs), ELCs were radioresistant and capable of self-renewal. However, despite their progenitor-like phenotype and expression of T cell lineage markers within the population, ELCs did not differentiate into conventional T cells or DETCs in *in vitro*, *ex vivo* or *in vivo* differentiation assays. Finally, we show that ELC expressed NK markers and secreted IFN-γ upon stimulation. Therefore we report the discovery of a unique population of lymphoid cells within the murine epidermis that appears related to NK cells with as-yet-unidentified functions.

The process of T cell differentiation from hematopoietic precursors has been studied for many years and is relatively well defined. Precursors leave the adult bone marrow (BM), or foetal liver in the case of embryonic T cell development, and arrive in the thymus as lymphoid progenitor cells. These progenitors proliferate and populate the thymus with immature thymocytes, which lack expression of the mature T cell markers CD3, CD4 and CD8 1,2. The dual absence of CD4 and CD8 during this phase characterizes the cells as “double-negative” (DN) T cell precursors, and their DN status is maintained through four further stages of differentiation named DN 1–4 1,2.

Movement through the DN stages is accompanied by progressive rearrangement of three out of the four T cell receptor (TCR) loci: γ, δ and β. If the TCRβ rearrangement is productive, it permits expression of the TCRβ chain, complexed with the germline-encoded invariant pre-TCRα (pTα)^3^,^4^,^5^,^6^. Upon expression of the TCRβ/pTα complex, called the pre-TCR, immature thymocytes are licensed to proliferate and rapidly progress to a CD4^+^CD8^+^ double-positive (DP) stage^7^. At this time, rearrangement of the TCRα locus takes place, resulting in expression of mature αβTCR complexes on DP thymocytes. Then DP thymocytes are subjected to positive and negative selection, which will result in death of 95% of thymocytes^7^. During this selection process, surviving thymocytes begin to down-regulate either CD4 or CD8 expression to become single-positive (SP) CD4^+^ or CD8^+^ thymocytes ready for export from the thymus to the periphery as naïve T cells^8^.

While this is clearly the route followed by the vast majority of T cells, it has recently become apparent that other mechanisms of generating specific T cell populations do exist, both prior to the development of the thymus in the foetus and independent of the thymus after birth. Rodewald *et al*. first identified a pro-thymocyte in murine foetal blood at a pre-thymic stage of development^9^, and later work described a phenotypically similar population in the adult mice, defining them as Committed T-cell Progenitors (CTPs)^10^. The CTPs, which are thought to be the precursors of the more-recently characterised Committed Intermediate Progenitors (CIPs)^11^, were first detected as an early T cell progenitor in adult murine BM that had the potential to give rise to mature T cells by an extra-thymic pathway^12^. Consistently, Nude (*Foxn1*-deficient) mice lack a functional thymus, yet retain some capacity to generate T cell populations in the mucosae^13^. Even within the populations of T cells that are generated within the thymus during embryonic development, some sub-populations appear capable of thymus-independent self-maintenance in the adult: the skin is a particularly interesting site in this regard. Murine Dendritic Epidermal T Cells (DETCs) are CD3^+^ T cells characterized by expression of Thy1 (Thymocyte antigen 1) and a unique γδ TCR comprising Vγ3/Jγ1-Cγ1 and Vδ1/Dδ2/Jδ2-Cδ chains^14^,^15^,^16^,^17^. DETCs are the first TCR-bearing cells to be generated in the foetal thymus, as early as embryonic day 14 (E14.0) of the 20 days of murine gestation^18^,^19^. These cells, expressing CD3 and the Vγ3/Vδ1 TCR, then expand, mature, and migrate to the skin as early as E16.0^19^,^20^,^21^. Within the epidermis, the unique TCR from DETCs is thought to recognize a putative self-antigen on damaged, stressed, or transformed keratinocytes^22^,^23^, and this population of T cells has now been shown to have roles in tissue and wound healing^24^,^25^,^26^. Early work on the properties of DETCs uncovered their ability, unusual amongst T cells, to maintain themselves locally, independently from blood circulating precursors^19^,^20^. Upon irradiation, the DETC population restores itself without input from any circulating or transplanted BM progenitor^27^. However, the exact mechanisms underlying the renewal of the DETC population are unknown, and the question of whether a DETC progenitor exists in the adult murine epidermis remains without answer.

Here, we used a combination of immune -sufficient and -deficient mouse models to reveal a further layer of complexity within the epidermal lymphoid compartment. We identified a novel population of lymphoid cells, named Epidermal Lymphoid Cells (ELCs), that appears in the epidermis during late embryogenesis and declines after birth in wild-type (WT) animals, but accumulates in mice with either extrinsic or intrinsic defects in T cell generation. Intriguingly, while these cells exhibit a phenotype suggestive of a T cell progenitor, *in vivo*, *ex vivo* and *in vitro* assays suggest they lack the capacity to proceed further in the T cell differentiation pathway and thus may represent a distinct sub-lineage. Expression of specific NK markers and cytokine production profile points towards ELCs to be related to the NK lineage, however the exact nature of this cell population and their potential immune functions remains to be revealed.

## Results

### Murine epidermis contains a population of Thy1^+^ cells that are distinct from DETCs

**Figure 1:**
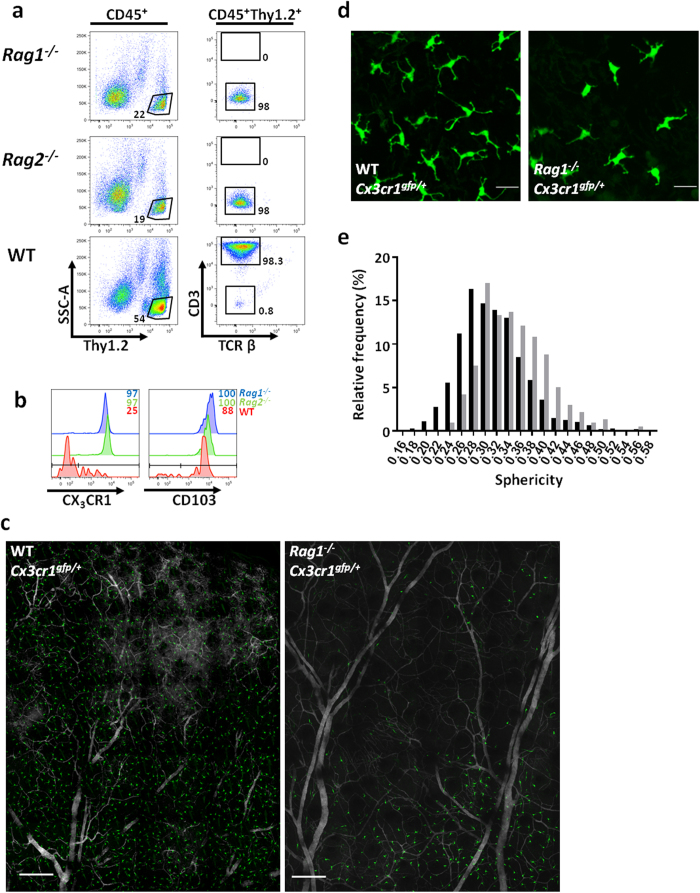
Thy1^+^ cells are present in the *Rag1*^−/−^ epidermis. (**a**) Flow cytometry of adult mouse epidermal cell suspension. Gating strategy to identify a Thy1^+^CD3^*−*^ population in both WT and *Rag-*deficient models is shown. (**b**). Histograms show relative expression intensity of CX_3_CR1 and CD103 among Thy1.2^+^CD3^*−*^ cells. Representative data from n > 5 mice is shown for a–b. (**c–d)** Two-photon imaging of the epidermis of WT and *Rag1*^−/−^*Cx3cr1*^*gfp*/+^ mice. In green are the CX_3_CR1-positive cells, scale 200 μm (**c**) and 20 μm (**d**). (**e**) Cell morphology analysis of CX_3_CR1-positive cells from WT (black) and *Rag1*^−/−^ (grey) mice. Representative data of n = 3.

Mice deficient in mature T and B cells through genetic ablation of either Recombination-Activating Gene (*Rag*) -1 or -2, completely lack DETCs in their epidermis^28^,^29^ (Fig. 1a). Here, the absence of DETCs in 8 week old (wo) *Rag1*- and *Rag2*-deficient mice revealed the existence of a minor population of Thy1^+^ cells in the epidermis that, unlike DETCs, did not express either CD3 or TCRβ (Fig. 1a). Detailed examination of epidermal suspensions from WT mice confirmed the presence of a minor population of these cells, representing approximately 1% of the CD45^+^Thy1^+^ population, which similarly lacked CD3 or TCRβ expression (Fig. 1a).

Phenotypic characterisation of the minor Thy1^+^ epidermal population from WT, *Rag1*^−/−^ and *Rag2*^−/−^ mice revealed uniformly high expression of the integrin CD103 (Fig. 1b), a marker normally associated with intraepithelial T cells^30^,^31^,^32^. Using the *Cx3cr1*^*gfp*/+^ reporter model^33^, our data revealed that the fractalkine receptor was expressed by 100% of the epidermal Thy1^+^ population from both *Rag*-deficient models, while the Thy1^+^CD3^*−*^ population from WT epidermis expressed varying levels of CX_3_CR1 and was restricted to only 25% of the total cells (Fig. 1b).

We also investigated the physical distribution of the epidermal Thy1^+^ cells in *Cx3cr1*^*gfp*/+^ mice. *Rag*-sufficient mice exhibited a uniform network of GFP^+^ cells since DETCs express CX_3_CR1. However, despite the fact that *Rag1*^−/−^ mice lack DETCs, *Rag1*^−/−^*Cx3cr1*^*gfp*/+^ epidermis clearly contained a population of GFP^+^ cells (Fig. 1c). In addition to their patchy distribution, Thy1^+^ cells in the *Rag1*^−/−^ mice were more markedly spherical, compared to the highly dendritic morphology characteristic of DETCs in WT mice (Fig. 1d,e).

### Differential CD2 expression sub-divides the minor epidermal Thy1^+^ population

Having identified a minor Thy1^+^ population that is distinct from DETCs, we next explored the possibility that these cells were related to Innate Lymphoid Cells (ILCs). ILCs are a diverse group of cells that exists as 3 main sub-populations, all of which resemble lymphoid cells in terms of morphology but lack the expression of a mature TCR^34^,^35^. Within the murine dermis, a population of ILC2 has been identified, which exhibits a characteristic phenotype, expressing Thy1 and ICOS (Inducible T-cell COStimulator), while lacking expression of CD3 and the inter-cellular adhesion/T cell activation molecule CD2^36^.

**Figure 2:**
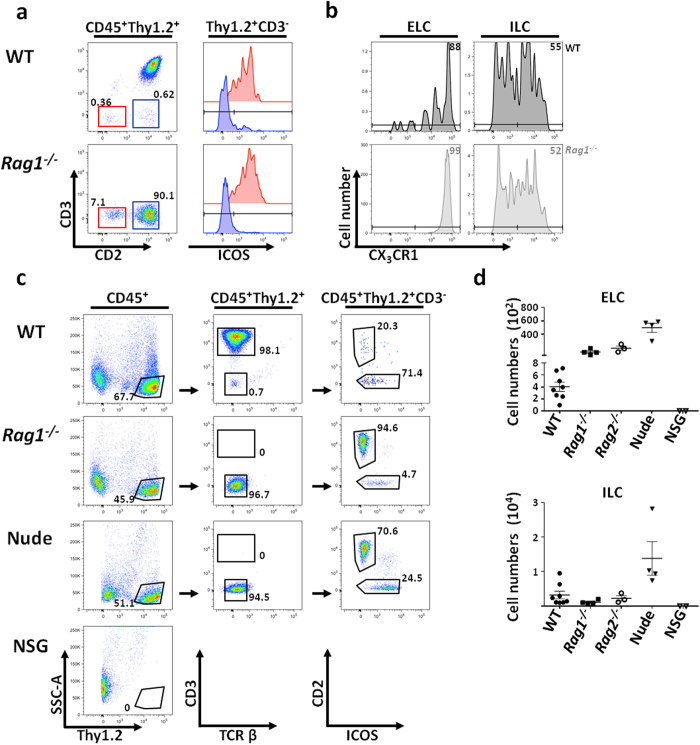
The epidermal Thy1^+^ population is heterogeneous. (**a**) Flow cytometry of mouse epidermal cell suspension. Gating strategy and histograms to identify ICOS expression across the two populations: Thy1^+^, ILCs (CD3^*−*^CD2^*−*^, red) and ELCs (CD3^*−*^CD2^+^, blue) in both WT and *Rag*-deficient models is shown. (**b**) Histograms show relative expression intensity of CX_3_CR1 among ELCs and ILCs from WT and *Rag1*^−/−^ mice. Representative data from n > 3 mice is shown a–b. (**c**) Identifi**c**ation of ELCs and ILCs in different mouse models. **c.** Absolute numbers of ELCs and ILCs in different mouse models. Representative data from n > 3 is shown, except for NSG where n = 2.

Using the same gating strategy as Roediger *et al*.^36^ (Fig. S1), we identified both CD2^+^ (blue) and CD2^*−*^ (red) cell populations within the Thy1^+^CD3^*−*^ epidermal compartment of *Rag1*^−/−^ and WT mice (Fig. 2a,b). The CD2^*−*^ sub-population also expressed ICOS, and therefore was likely ILC2, while the CD2^+^ sub-population of the minor epidermal Thy1^+^ cells was ICOS^*−*^ (Fig. 2a) and expressed high levels of CX_3_CR1 in both WT and *Rag1*^−/−^. The ILC compartment had a heterogeneous profile of expression for the same fractalkine receptor (Fig. 2b). Thus, the majority of the murine epidermal CD3^*−*^Thy1^+^ population, which expresses CD2 and lacks ICOS, is distinct from both DETCs and ILCs. They represent a novel and discrete population that we named Epidermal Lymphoid Cells (ELCs).

The genetic ablation of the *Rag* genes causes an intrinsic defect in T and B cell maturation in the context of otherwise normal immune anatomy^28^,^29^. In contrast, the Nude mouse model is rendered effectively athymic by a mutation in the *Foxn1* gene that results in defective development of the thymic epithelium and the almost complete absence of T cells^37^,^38^. Interestingly, we also detected a population of Thy1^+^CD3^*−*^ cells in the epidermis of Nude mice, which, similar to the *Rag1*^−/−^ and WT, contained both ELCs and ILCs (Fig. 2c,d). Therefore, we conclude that ELCs arise independently of classical thymus-dependent pathways of differentiation.

Given the thymic-independence of ELC generation, we asked which other components of the immune differentiation pathways were these cells reliant upon. The common gamma chain (γ_c_), also known as IL-2R gamma (IL-2Rγ), is a cytokine receptor subunit that is part of the receptor complexes for at least six different interleukin receptors: IL-2, IL-4 IL-7, IL-9, IL-15 and IL-21^39^,^40^,^41^,^42^,^43^,^44^,^45^. As a consequence, the *γ*_*c*_-deficient mouse model, such as NOD *scid* gamma (NOD/SCID/γ_c_ null or NSG), lack all mature T cells^46^, including DETCs^47^. Hence, we tested if ELC were also γ_c_-dependent. Examining the epidermis of NSG mice revealed the complete absence of Thy1^+^ cells, including ELCs. Hence while ELCs are not dependent on the thymus for their derivation, they do require γ_c_ signaling for either their differentiation, survival or maintenance.

### ELCs are radioresistant and possess self-renewal capacity

**Figure 3:**
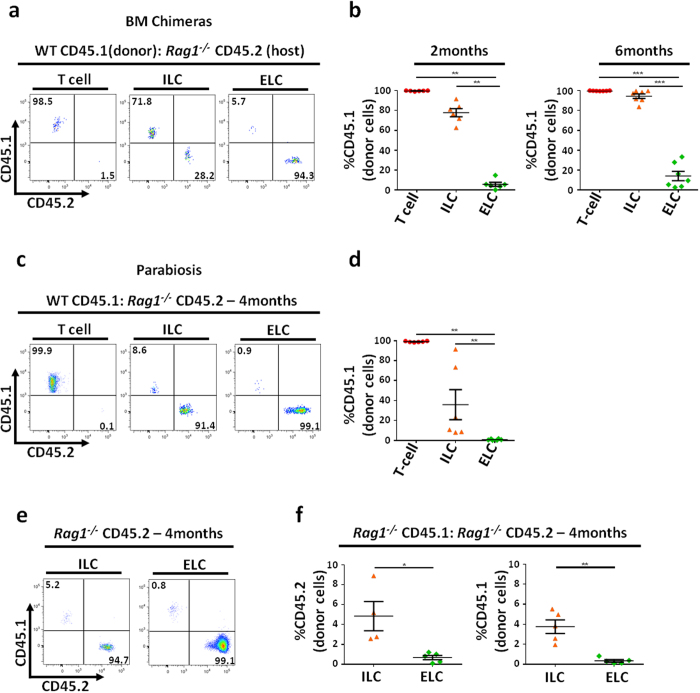
ELCs cells are radioresistant and renew themselves locally. (**a**–**b**) Flow cytometry analysis showing percentage of CD45.2 and CD45.1 T cells, ILCs and ELCs in mouse epidermal cell suspension from BM chimeric mice (CD45.1 WT: CD45.2 *Rag1*^−/−^) (2 months n = 6; 6 months n = 7). (**c**–**d**) WT CD45.1 mice were joined surgically with CD45.2 *Rag1*^−/−^ mice to create parabiont pairs. 4 months later flow cytometric analysis was performed on the *Rag1*^−/−^ epidermal cell suspensions. n = 4. (**e**–**f**) CD45.1 *Rag1*^−/−^ mice were joined surgically with CD45.2 *Rag1*^−/−^ mice to create parabiont pairs. 4 months later flow cytometric analysis was performed on epidermal cell suspension from both mice (n = 4).

Unlike most immune cell populations, which are derived from the BM, within the epidermis, both DETCs and Langerhans cells (LCs), are resistant to depletion by irradiation and renew themselves locally^27^,^48^,^49^. Using BM chimeras, we asked whether the same was true for the ELC population. We lethally-irradiated CD45.2 *Rag1*^−/−^ host mice before infusing flushed BM cells from CD45.1 WT donors. After 2 months, 99.8 ± 0.1% T cells and 77.8 ± 4.1% of ILCs were of donor origin (Fig 3a,b), in agreement with published data^36^, while most ELCs were still of host origin. By 6 months post-reconstitution, ILCs were almost exclusively of donor origin (Fig. 3b), while 86 ± 4.6% of ELCs remained of host origin. Thus, like DETCs and LCs in the epidermis, ELCs are radioresistant and do not undergo replenishment from the BM cell pool following irradiation.

While BM chimeras are often informative, the process of irradiation and reconstitution results in an inflammatory environment that may introduce experimental artefacts^50^. Therefore we also assessed the homeostatic turnover of epidermal cell populations using parabiotic mice. In this model, two adult congenic mice differing in expression of CD45 alleles are surgically attached in order to link their circulatory systems. This technique enables measurement of the extent of the contribution made by blood-borne cells from each parabiont to the immune cell populations in the other, over prolonged periods and without the need for irradiation. We joined congenic WT CD45.1 and *Rag1*^−/−^ CD45.2 mice and assessed the cellular origin of the CD45^+^ hematopoietic cell compartment in the epidermis of the *Rag1*^−/−^ mice 4 months later. As expected from the inability of *Rag1*^−/−^ mice to produce their own T cells, all CD3^+^ cells in the *Rag1*^−/−^ epidermis were of donor origin, while ILCs exhibited marked heterogeneity in source (Fig. 3c,d). In contrast, 99.1 ± 0.3% of ELCs remained of host origin (Fig. 3c,d). To exclude the possibility that WT mice, which bear fewer ELCs than their *Rag1*^−/−^ counterparts (Fig. 1a), were simply unable to provide the appropriate blood-circulating precursors for ELCs, we confirmed our findings in *Rag1*^−/−^ CD45.1*/Rag1*^−/−^ CD45.2 parabionts. Similarly, after 4 months of parabiosis, ELCs in the epidermis of both mice remained of host origin (Fig. 3e,f). In summary, ELCs, similar to DETCs, are epidermal-resident cells that do not undergo replenishment from precursors arising from BM or borne in the blood, either following irradiation or during prolonged periods of parabiosis.

### ELCs express heterogeneous levels of T lineage markers

**Figure 4:**
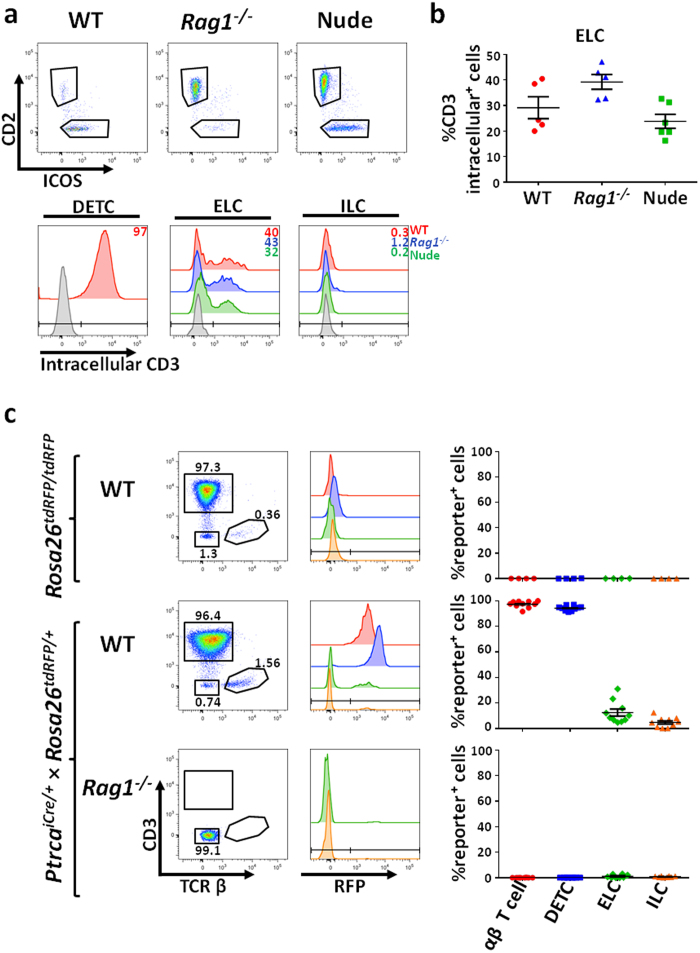
ELCs have a T cell progenitor-like phenotype. (**a**) Flow cytometry of mouse epidermal cell suspensions with intracellular labelling for CD3. Histograms show the expression profile of intracellular CD3 for DETCs, ELCs and ILCs in several mouse models. (**b**) Plot shows the percentage of ELCs from WT (red), *Rag1*^−/−^ (blue) and Nude (green) mice that express CD3 intracellularly. n = 5 for WT and *Rag1*^−/−^ mice, n = 6 for Nude mice. (**c**) Gating scheme to identify pTα expressing cells from the mouse epidermis. The histograms and dot plots show the frequency of current or past pTα expression within the αβT-cell (red), DETC (blue), ELC (green) and ILC (orange) populations. Each data point represents an individual mouse. Data were obtained from n = 4 of Rosa26^*tdRFP/tdRFP*^ control mice, Ptrca^*icre*/+^ x Rosa26^*tdRFP*/+^ n = 10 on a WT background and n = 8 on a *Rag1*^−/−^ background.

Since ELCs share several characteristics with DETCs and also express lymphoid markers, we wondered whether they might represent local DETC precursors. Several studies have shown that T cell progenitors in the adult or embryonic thymus express CD3 intracellularly^51^,^52^, and that cytoplasmic CD3 might be considered a hallmark of the T cell lineage^53^. Therefore, we investigated whether ELCs expressed CD3 in their cytoplasm. Intracellular expression of CD3 in the ELC population varied from 25–40% in WT, *Rag1*^−/−^ and Nude mouse models, while ILCs were devoid of cytoplasmic CD3 (Fig. 4a,b). Having suggestive properties of a T cell identity, as intracellular CD3 expression was heterogeneous within the ELC population, we next extended our phenotypic analysis to other markers of the T cell lineage.

The pre-TCRα (pTα) chain is an essential and invariant subunit of the pre-TCR, whose known physiological function is to associate with nascent TCRβ chains in T lineage committed progenitors^54^. Accordingly pTα is almost-exclusively expressed by immature T cell precursors from both thymic and and extra-thymic T cell development pathways^55^. Therefore, we employed a fate-mapping mouse model expressing CRE recombinase under the control of the endogenous pTα-encoding gene locus (*Ptcra*) enabling all cells that have expressed pTα to be heritably marked^56^. To trace the progeny of pTα expressing cells, we crossed *Ptcra*^*iCre*^ knockin mice with *Rosa26*^*tdRFP*^ reporter line to produce a model in which all cells expressing pTα or deriving from pTα-expressing cells express RFP. As negative control, *Rosa26*^*tdRFP/tdRFP*^ mice lacking iCre expression were used (Fig. 4c). In 8 week-old mice, all αβ T cells in the skin have history of pTα expression, as expected^56^, as did the vast majority of DETCs (97 ± 0.8%)(Fig. 4c). In contrast, few cells with an ILC-like phenotype expressed pTα, 4.8 ± 1.2% (Fig. 4c), and the ELC population exhibited a heterogeneous profile of expression (12 ± 2.7%) (Fig. 4c). These data suggest that ELCs are unlikely to represent a homogeneous population of DETC progenitors; and that the heterogeneity of T-lineage molecule expression within the ELC population may reflect the presence of distinct maturation stages of ELC, with some belonging to a stage before pTα expression. In agreement with the latter, ELCs from *Ptcra*^*iCre*^ knockin mice with a *Rag1*^−/−^ background crossed with the *Rosa26*^*tdRFP*^ reporter line were not RFP-labelled in this model, leading us to speculate that the absence of *Rag* expression limits the ability of ELCs to develop normally. Taken together, the observed degrees of expression of intracellular CD3 and pTα across ELCs in the WT background mice led us to hypothesise that the ELC population might comprise a heterogeneous pool of T-lineage-committed cells.

### Adult ELCs do not differentiate into T cells

**Figure 5:**
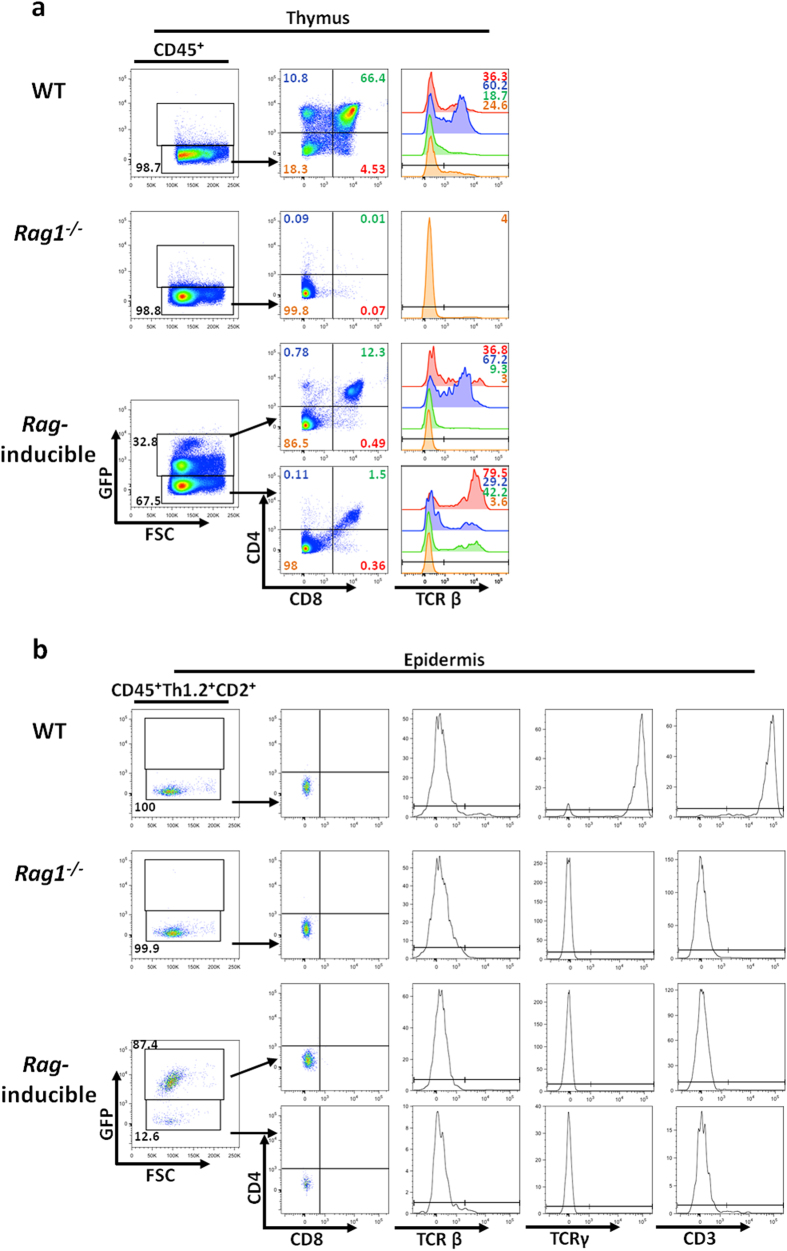
ELCs do not develop into mature T cells upon *Rag* re-expression. WT, *Rag1*^−/−^ and Rag inducible model were injected i.p. with tamoxifen daily for 5 days and tissues were analysed 30 days later by flow cytometry. GFP signal indicates *Rag* recombination and expression. Flow cytometry of mouse thymus (**a**) and epidermis (**b**) cell suspensions from WT and *Rag1*^−/−^ controls, and the *Rag* inducible mouse model. Data are representative of n = 3.

The fate mapping data showed that some of the ELCs from WT mice had differentiated sufficiently far down the T cell pathway to express pTα. However, this was not the case in *Rag1*^−/−^ mice, perhaps because the lack of *Rag* expression created an intrinsic block at a stage preceding pTα expression. To attempt to rescue ELC development in *Rag1*^−/−^ mice, we developed a *Rag* inducible model (Fig. S2 and Methods). DNA fragment containing 2A peptide sequences between *Rag1* and *Rag2* cDNA after the loxP flanked Stop cassette was inserted into the *Rosa26* locus of WT Embryonic Stem (ES) cells. The resulting mouse model was crossed to *Rag1*^−/−^ Rosa26^Cre-ERT2*/+*^ (Ref. 57), resulting in *Rag1*^+/−^ mice harboring an inducible *Cre-ERT2* transgene or the *stop-Rag1A2* fragment in their *Rosa26* locus, respectively. Mice were then further intercrossed to obtain the desired genotype, the *Rag*-inducible model on *Rag1*^−/−^ background. Upon tamoxifen administration that activates the Cre recombinase, these mice will produce both Rag1 and Rag2 protein from *Rosa26*^*Rag1A2*^ allele as well as express GFP as a reporter of recombination (Fig. S2 and Methods), allowing identification of *Rag*-expressing cells by flow cytometry (Fig. 5). We hypothesized that by inducing expression of the *Rag* genes that the cells were missing, the developmental potential of ELC would be restored, with the possibility of proceeding from their double negative (DN) CD4^*−*^CD8^*−*^ status to the DP^4^, or subsequent single positive CD4 (CD4^+^CD8^*−*^, SP CD4^+^) or CD8 (CD4^*−*^CD8^+^, SP CD8^+^) stages^8^.

*Rag* gene expression in our inducible model was induced by intraperitoneal administration of Hydroxytamoxifen (4’OHT) over 5 consecutive days. One month later, both thymus and epidermis from WT, *Rag1*^−/−^ and *Rag*-inducible mice were collected and their cell populations analysed by flow cytometry. Approximately one third of thymocytes in the *Rag*-inducible model expressed GFP, and 12% of these thymocytes had proceeded to the DP stage, while all thymocytes in the *Rag1*^−/−^ were arrested at the DN stage (Fig. 5a). Furthermore, in the *Rag*-inducible mice, 0.5–1% of thymocytes achieved SP expression of CD4 or CD8 with TCRβ expression (Fig. 5a), showing that our rescue model was able to partially overcome the blockade to thymic T cell development seen in *Rag1*^−/−^ mice. However, in the epidermis of the same mice, ELCs exhibited comparable phenotypes regardless of the induction of *Rag* expression (Fig. 5b). Even upon reinstatement of the *Rag* genes, no change in expression of markers linked to T cell maturation, such as the TCR or CD3, was observed. Later time points and topical delivery of 4’OHT revealed similar results (*data not shown*). Hence, ELCs either are not T cell progenitors or cannot differentiate in the adult skin environment.

### ELCs are present in the murine epidermis prior to birth

**Figure 6:**
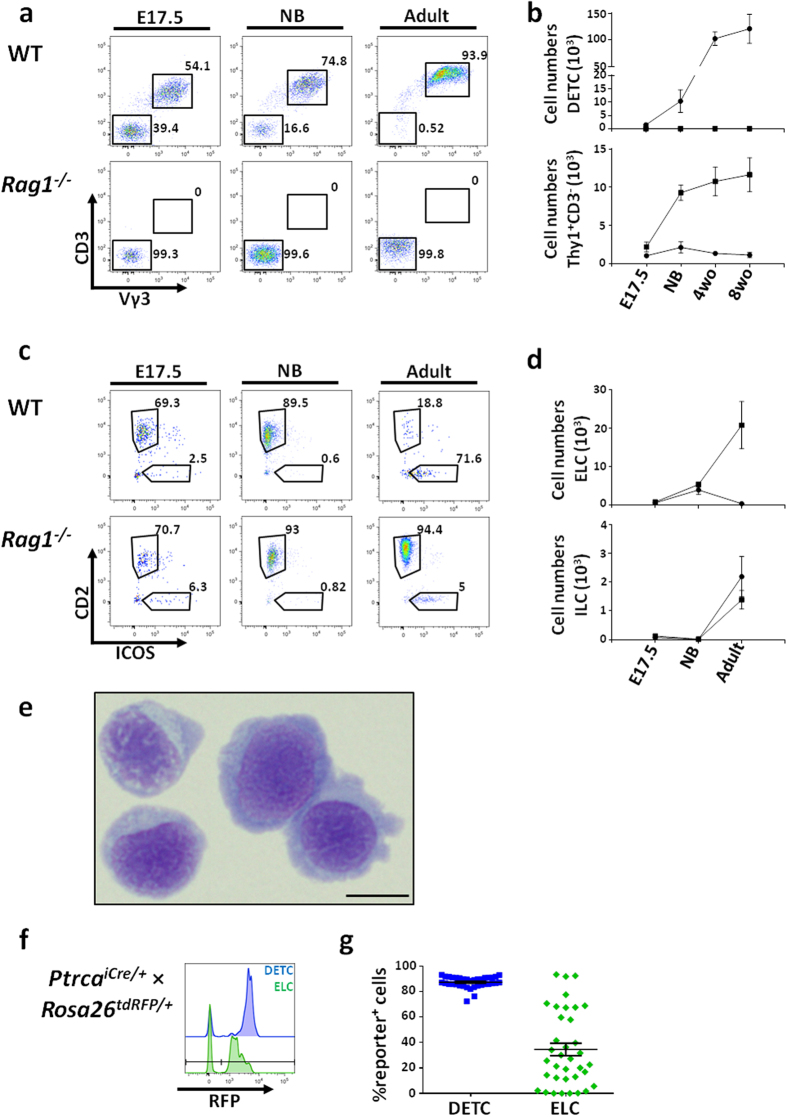
ELCs are present before birth in both WT and *Rag1*^−/−^ mice. (**a**) Flow cytometry of mouse epidermal cell suspensions. Gating strategies for DETC and Thy1^+^CD3^*−*^Vγ3^*−*^ populations are shown throughout mouse development (among CD45^+^Thy1^+^ cells). (**b**) Absolute numbers of both populations before birth, in newborns and in adult WT (•) and *Rag1*^−/−^ (■) mice. Representative data from n > 3. (**c**) Dot plots show the gating scheme revealing the heterogeneity of the Thy1^+^CD3^*−*^Vγ3^*−*^ population, which is mainly composed of ELCs, in the mouse epidermis. (**d**) Absolute numbers of ELCs and ILCs, before birth, in newborns and in adult WT (•) and *Rag1*^−/−^ (■) mice. Representative data from n > 3 (**e**) Purified neonatal ELCs were spun onto cytospin slides for Wright-Giemsa staining (Scale bar of 10 μm). Data are representative of 3 independent experiments. (**f–g**) Plots identify cells with a history of pTα-expression in E18.5 epidermis. The histograms and dot plot identify pTα-expressing cells within the DETC (blue) as well as ELC (green) populations. Each data point represents an individual embryo. Data were obtained from n = 36 embryos from 6 independent experiments of *Ptrca*^*icre*/+^ x Rosa26^*tdRFP*/+^ mice.

DETCs arise from embryonic precursors and self-maintain into adulthood^18^,^19^,^27^. Given the homeostatic similarities we uncovered between ELCs and these cells, we asked whether ELCs might also seed the skin prior to birth. In the WT E17.5 epidermis (the earlier time point to separate dermis from epidermis), we detected a population of CD3^+^ cells that were confirmed as DETCs according to expression of the Vγ3 TCR, and in agreement with early reports^19^. However, alongside DETCs, a population of Thy1^+^CD3^*−*^Vγ3^*−*^ cells was present in both WT and *Rag1*^−/−^ embryos (Fig. 6a). Furthermore, the relative frequency of this Thy1^+^CD3^*−*^Vγ3^*−*^ population increased in the *Rag1*^−/−^ epidermis similarly to what we observe in WT mice with the DETC compartment (Fig. 6b). To further characterize this Thy1^+^CD3^*−*^Vγ3^*−*^ population in terms of their relation to ILCs, we measured expression of CD2 and ICOS. Before birth, the majority (70%) of the Thy1^+^CD3^*−*^Vγ3^*−*^ cells in the WT and *Rag1*^−/−^ epidermis were CD2^+^, likely corresponding to the ELCs observed in the adult (Fig. 6c). At the newborn (NB) stage, the number and frequency of ELCs increased in both groups of mice, but while this increase was maintained into adulthood in the *Rag1*^−/−^, in the WT animals the ELC population decreased dramatically (Fig. 6d). In addition, Wright-Giemsa staining showed that neonatal ELCs were small in size and had a round shape, dark nucleus and scanty cytoplasm (Fig. 6e) - all characteristics of lymphoid cells. Of note, we did not find ELC-like populations in tissues (lung, liver, gut, kidney) other than neonatal epidermis (*data not shown*).

Having established the presence of ELCs in the embryos of both WT and *Rag1*^−/−^ mice, we again asked whether these cells were bound to the T cell lineage prior to birth (Fig. 6f, g). In the E18.5 embryos from the pTα fate mapping model used previously, we observed that 87.2 ± 0.7% of DETCs exhibited a history of pTα-expression, while ELCs were again heterogeneous for prior or current pTα expression (average 34.5 ± 5%). These findings further support the notion that the ELC population might be a heterogeneous pool of cells, some of which are committed to the T cell lineage, while some remain T cell-uncommitted.

### Neonatal ELCs do not differentiate into T cells

**Figure 7:**
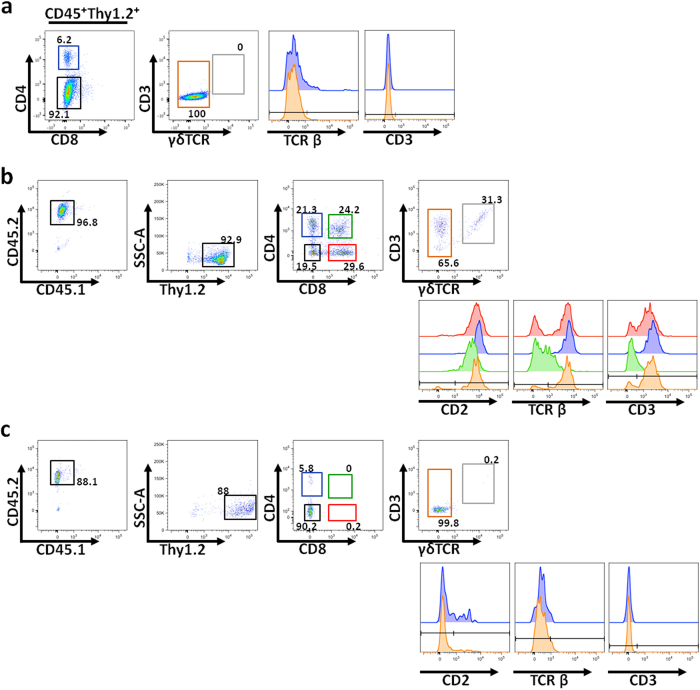
Neonatal ELCs do not develop into mature T cells. (**a**) Progeny of ELC co-culture with TSt-4 stromal cells after 9–12 days were analysed for expression of maturation markers of the T cell lineage, including CD4, CD8, CD3 and TCR. n = 6. (**b**–**c**) Progeny of the thymic DN cells (**b**) and ELCs (**c**) after culture in foetal thymic organ culture for 12–15 days, analysed for CD4 and CD8 expression by flow cytometry. Data are representative of n = 3.

We hypothesized that the skin might not provide the adequate environment for these cells to further develop along the T cell pathway. Hence we tested if the ELCs had T cell differentiation potential *in vitro*. We purified ELCs from WT NB epidermis and co-cultured them with TSt-4 stromal cells expressing Delta-like 1 (TSt4/DL1), a foetal thymic fibroblastoid cell line commonly used in T cell differentiation assays^58^. In addition, a cytokine cocktail including IL-2, IL-7 and IL-15 was also added to the culture to reveal any inherent potential of the ELCs to develop towards a more mature phenotype^58^. However, after 9–12 days co-culture in the cytokine-enriched stromal environment, ELCs maintained their CD3^*−*^ TCR^*−*^ phenotype, and did not differentiate into T cells or more mature T cell progenitors (Fig. 7a).

We also tested the T cell potential of neonatal ELCs using foetal thymic organ cultures (FTOCs), an *ex-vivo* system that supports the complete T-cell development program, including positive and negative selection steps^59^. We cultured E15.5 thymic lobes from CD45.1 WT mice with 2’-deoxyguanosine (dGuo) for 7 days selectively depleting their hematopoietic compartment. At this time, ELCs and thymic DN cells (positive control) were isolated from WT NB mice (Fig. S3). Purified cell populations were added to the hematopoietic cell-depleted lobes and cultured for 12 to 15 days. After this time, the thymic DN cells had developed as *in vivo*: both DP and SP stages were evident, with abundant expression of TCRβ and CD3 (Fig. 7b). Some γδ T cells were also detected. In contrast, ELCs in FTOC maintained their Thy^hi^ profile, down-regulated CD2 expression, and did not express TCR, CD3 or CD8 (Fig. 7c). While approximately 5% of cultured ELCs did express CD4, as their *in vivo* counterpart (data not shown), they did not exhibit any other aspects of late DN, DP or SP thymocyte stages (Fig. 7c). Therefore, we conclude that neonatal ELCs are not T cell progenitors.

### Neonatal ELCs have a NK-like phenotype

**Figure 8:**
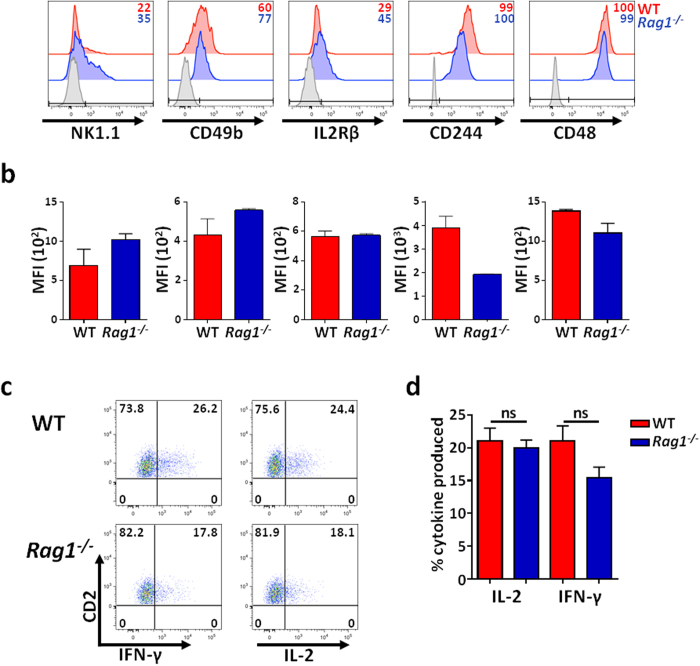
Neonatal ELCs express NK cell marker and secrete IFN-γ and IL-2. (**a**) Representative histograms of the level of expression of NK markers by ELCs present in epidermal cell suspensions of NB from both WT and *Rag1*^−/−^ models. (**b**) Mean Fluorescence Intensity (MFI) with standard deviation (SD) bars of NK marker expression by ELC population from both WT and *Rag1*^−/−^ mice. Data representative n = 3. (**c**–**d**) Representative dot plots and bar graphs of the production of IFN-γ and IL-2 from ELCs from WT and *Rag1*^−/−^ NB after 4 h stimulation with PMA and ionomycin. Data representative of n = 3.

Both CD2 and Thy1 markers are not restricted to the T cell lineage since expressed by other cell types, including NK cells^60^. Therefore, we hypothesized that the ELC population could be related to NK cells. Accordingly, both WT and Rag1^−/−^ ELC populations expressed low levels of CD49b and had a partial expression of NK1.1 (Fig. 8a,b), specific markers found on the surface of murine NK cells. In addition, low levels of IL-2Rβ were also found in neonatal ELCs of both mouse models (Fig. 8a,b). Interleukin 2 (IL-2) is a major growth factor for mature NK cells^61^,^62^. Freshly isolated NK cells preferentially express IL-2Rβ, through which IL-2 plays a pivotal role in proliferation and induction of cytolytic activity^63^. In addition, the CD2/SLAM family includes several members such as CD2, 2B4 (CD244) and CD48. Murine CD244 is expressed on different subsets of cells, such as T cells and all NK cells^64^,^65^,^66^. In addition, CD48 is expressed widely on hematopoietic cells including T cells and NK cells and has been identified as a ligand for CD244 and CD2^66^. Interestingly, neonatal ELCs expressed both CD244 and CD48 in the WT and Rag1^−/−^ models (Fig. 8a,b).

IFN-γ secretion after simulation is a hallmark of NK cells^67^. Hence, we stimulated NB epidermal cell suspensions *in vitro* and tested their capacity to secrete IFN-γ as well as other relevant cytokines. After stimulation with PMA plus ionomycin, around 20% of ELCs from both WT and Rag1^−/−^ expressed IFN-γ (Fig. 8c,d). Similar percentage of ELCs expressed IL-2 as well, a cytokine crucial for NK differentiation and function^61^,^62^. Production of IL-4, IL-5, IL-9, IL-10, IL-13, IL-17, IL-22, TNF-α, granzyme and perforin were also analysed, revealing that ELCs did not produce any of them with or without stimuli (*data not shown*).

## Discussion

Cutaneous lymphoid populations have been extensively studied under both steady state and inflammatory conditions. DETCs are the major lymphoid cell type in the murine epidermis in the steady state^14^, while following inflammation epidermal CD8^+^ T cells become part of the immune pool found in this tissue^68^. In contrast the dermis is populated by different types of T cells including αβ and γδ T cells, NK cells and the recently-described ILC2^36^,^69^,^70^. Here we identified a new lymphoid population that is present at low frequencies in the adult WT epidermis, but accumulates in the epidermis of adult *Rag*-deficient models and athymic mice - the Epidermal Lymphoid Cells (ELCs).

ELCs share several homeostatic features with DETCs, including radioresistance and independence from circulating progenitors for their maintenance. Considering these similarities and the expression of lymphoid markers by ELCs, we first hypothesized that they could represent DETC local progenitors, belonging to the T cell or T cell progenitor family. It has been reported that cells belonging to T cell lineage express CD3 intracellularly^51^,^52^,^53^, and variable proportions of the ELC population were positive for cytoplasmic CD3. Moreover, prior or current expression of the T lineage-restricted marker pTα was detected in some ELCs as well as all DETCs. Taken together, the expression of CD3 and pTα by some cells within the ELC population is indicative of a T cell lineage identity. ELCs also expressed CD2, leading us to hypothesize that these cells might be related to the CIPs described by Dejbakhsh-Jones and collaborators^10^. If true, these intermediate progenitors should be able to develop through an extrathymic pathway into mature T cells. However, using a *Rag-*inducible mouse model that enabled reinstatement of normal thymocyte development, ELCs retained their existing phenotype. One possible explanation is that the adult skin simply does not provide the correct environment for these cells to further differentiate: maybe, similarly to DETCs^20^, ELCs are restricted to a specific time line during development. In order to understand their true developmental potential we isolated neonatal ELCs and attempted to reveal their differentiation capacity via *in vitro* and *ex vivo* approaches. In co-culture with TSt4/DL1 cells or FTOC cultures, ELC did not express T cell maturation markers such as CD3 or TCR, but maintained the same phenotype throughout the culture period. Hence, despite exhibiting T lineage characteristics in both adult and foetal skin, ELC might not belong to the T cell lineage or represent peripheral T cell progenitors.

The true nature of ELCs remains unknown. As lymphoid-like cells, they could be related to ILC. However, their phenotype is different from *bona fide* ILC, lacking expression of surface markers characteristic of the different ILC groups such as ICOS, NKp46, ST2 and c-Kit (*data not shown*). More importantly, ELC are radioresistant, unlike the ILC2 described by Roediger^36^ and other ILC^71^,^72^. Accordingly, all the immune population resident of the epidermis, such as DETCs and Langerhans cells (LCs), are resistant to depletion by irradiation and renew themselves locally^27^,^48^,^49^. Alternatively, ELCs could be NK-like cells, which also express Thy1 and CD2 on their surface. However, neonatal ELCs only partially express NK classical markers such as CD49b and NK1.1. Another hallmark of NK cells is their rapid secretion of IFN-γ and IL-2 upon stimulation, exhibiting a Th1-like profile of cytokine^73^. Importantly, neonatal ELCs from both WT and *Rag1*^−/−^ are able to produce IFN-γ and IL-2, supporting their NK-like nature. Further studies regarding ELC function, transcription factor’ profile and dependency might provide further insights into their true nature.

ELCs were also present in E17.5 epidermis in WT and represent a significant population. Up to the NB stage, the frequency of ELCs was the same in both WT and *Rag1*^−/−^, and similar to the frequency of DETCs. However, in adult mice, ELCs were increasingly abundant in the *Rag1*^−/−^, while they became rare in the WT epidermis. Combined with the knowledge that ELCs are able to self-maintain locally independent of circulating progenitors, it is likely that embryonic ELCs that seed the skin prenatally give rise to the ELCs found in adulthood, in particular the population found in the *Rag1*^−/−^. Thus, we hypothesize that the disappearance of ELCs in WT postnatal epidermis might be due to direct competition with DETCs for niche occupation or cytokine requirements. IL-15 produced by keratinocytes has been reported to induce growth of the DETC population^47^. Thus, IL-15 arises as a possible candidate for the cytokine competition hypothesis drawn between DETCs and ELCs. Nevertheless, localization studies would have to be addressed in order to further understand the exact proximity of these two groups of cells that might lead to cell competition.

Finally, the specific time frame in which these cells are present in the WT epidermis, plus the expression of ligand/receptor CD48/CD244/CD2 (as CD244 and CD48 expression was been reported to be expressed by DETCs^74^ and LCs^75^ respectively) indicates a possible cooperative role for ELCs alongside LCs and DETCs in supporting the establishment of a mature epidermal immune system at birth. Of note that skin-resident DCs have recently been shown to have an active role in remodelling the skin microbiome^76^. Therefore, we hypothesize that these three immune compartments might cooperate on a tolerogenic role towards skin microbiota, but additional investigations would be required in order to further understand the mechanisms behind such collaboration. Additionally, other possible hypotheses would be that ELCs could interact with keratinocytes and be involved in the maturation mechanisms of LCs or could mediate the LC/DETC activation upon microbiome establishment. Thus the role and lineage relations of the ELC population remain an enigma at this stage and should prove a productive avenue for future research into immunity in the skin.

## Methods

### Mice

C57BL/6 (CD45.2) mice were purchased from the Biological Resource Center (BRC), Agency for Science, Technology and Research (A*STAR), Singapore. C57BL/6 (CD45.1), B6.Cg-*Foxn1*^*nu*^/J (Nude), B6.129S7-Rag1tm1Mom/J (*Rag1*^−/−^), B6.129P-Cx3cr1tm1Litt/J mice (*Cx3cr1*^*gfp*/+^), were purchased from the Jackson Laboratory (Jackson Laboratory, Bar Harbor, USA). B6.129S6-Rag2tm1Fwa N12 (*Rag2*^−/−^) were purchased from Taconic (Taconic Farms, USA). *Rag1*^−/−^ mice were crossed with C57BL/6 CD45.1 to give rise to congenic *Rag1*^−/−^ CD45.1 mice, or with *Cx3cr1*^*gfp*/+^ mice to generate *Rag1*^−/−^ *Cx3cr1*^*gfp*/+^ mice. All *Ptrcai^Cre/+^ × Rosa26^tdRFP/+^* mice were kindly provided by Dr. Hans Jörg Fehling (Institute of Immunology, University Clinics, Ulm, Germany). NOD *scid* gamma (NSG) mice were kindly provided by the National University of Singapore. All mice were bred and maintained in our animal facility, and adult mice were analysed between 8-12 weeks of age. All experiments and procedures were approved by the Institutional Animal Care and Use Committee of A*STAR (Biopolis, Singapore) in accordance with the guidelines of the Agri-Food and Veterinary Authority and the National Advisory Committee for Laboratory Animal Research of Singapore.

### Flow cytometry, cell sorting and intracellular cytokine staining

Flow cytometry was performed on a LSR II with 5 lasers, or an Aria II with 4 or 5 lasers (Becton Dickinson, San Jose, USA) and analysed with FlowJo software (Tree Star, Ashland, USA). Fluorochrome-conjugated monoclonal antibodies (mAbs) specific to mouse CD45 (30F11), CD45.1 (A20), CD45.2 (104), CD2 (RM2-5), CD3 (145-2C11), CD4 (GK1.5), CD8 (53-6.7), CD48 (HM48-1), CD49b (DX5), CD90.2 (53-2.1), TCRβ (H57-597), TCRγ/δ (GL3), Vγ3 (536), CD103 (2E7), CD244 (244F4), CD278 (C398.4A), NK1.1 (PK136), IL-2Rβ (TMb1), IL-2 (JES6-5H4), IFN-γ (XMG1.2) were purchased either from BD Biosciences (San Jose, USA) or Ebiosciences (San Diego, USA). Intracellular labelling for CD3, IL-2 and IFN-γ was performed on cells previously labelled for surface markers, following fixation and permeabilisation with Fix/Perm solutions (BD Biosciences, Mountain View, CA) according to the manufacturer’s instructions. Intracellular cytokine staining was performed on epidermal cell suspensions after stimulation for 4 hr with phorbol myristate acetate (PMA) (500 ng/mL) and ionomycin (500 ng/mL) (Sigma-Aldrich).

### Mouse skin cell preparation

Mouse skin cells were isolated as described previously^77^. Briefly, embryonic/neonatal skin was detached from the body or mouse ears were split into dorsal and ventral halves and floated on RPMI-1640 medium (Sigma) containing 1 mg/ml dispase (Invitrogen) for 60 min to allow separation of epidermal and dermal sheets. Epidermal and dermal sheets were then cut into small pieces and incubated in RPMI containing 10% feotal calf serum (FCS), 0.8 mg/ml collagenase type IV (Sigma) and 50 μg/ml DNAse I (Roche) for 90 min. Cells were then passed through 19 G syringe and filtered through a 70 μM cell strainer (BD Falcon) to obtain a homogenous single cell suspension. For purpose of cell number normalisation, analyses were performed on both ears of the adult mice and the corresponding area, 3 cm^2^, of the body skin from the embryos and newborn.

### Intravital multiphoton imaging of mouse ear skin

Mice were anaesthetized with a cocktail of 150 mg/kg ketamine and 10 mg/kg xylazine, before ear hair was removed with the depilatory lotion Veet. Anaesthetized mice were immobilized on a custom-made stage^78^, with a heating pad attached to maintain the animal at 37 °C. To label blood vessels *in vivo*, mice were retro-orbitally injected with 40 μl of a 10 mg/ml solution of Evans Blue dye (Sigma-Aldrich). Images were acquired using a multiphoton microscope system (LaVision Biotec) with a tunable Chameleon Ultra II Ti:Sapphire laser (Coherent) at 950 nm, and the following long pass mirrors and bandpass filters: 495 LPXR (Chroma), 560 LPXR (Chroma); 475/42 (Semrock), 525/50 (Chroma), 655/40 (Semrock). Data sets generated were analysed by IMARIS imaging software (Bitplane).

### Generation of bone marrow chimeras

Recipient 7 to 8 week old CD45.2 *Rag1*^−/−^ mice were lethally irradiated (2 × 600 rad, 3 hr apart using a Caesium source) and reconstituted by intravenous injection with CD45.1 WT BM. Engraftment was assessed by measuring the relative numbers of donor cells among blood CD45^+^ cells 4 weeks after transplantation. Epidermal cell suspensions from mice were analysed 2 or 6 months post-transplant and the proportion of DETCs, ELCs and ILCs derived from WT (CD45.1) cells was determined.

### Generation of parabiotic mice

CD45.2 *Rag1*^−/−^ mice were sutured to either CD45.1 WT or CD45.1 *Rag1*^−/−^ mice and remained together for 4 months. All mice were 5 to 6 weeks old at the time of surgery. Mice received ketamine/xylazine as an anaesthetic during surgery, plus buprenophine and baytril for analgesia for the few days following the procedure.

### Wright-Giemsa staining

For cytospin, purified cells were spun onto glass slides, fixed and stained using the Hema 3 System (Thermo Fisher Scientific), and rinsed in distilled water. Images were analyzed using an Olympus BX43F conjugated with an Olympus DP21 digital camera. Snapshots were taken at a 10 × 100-fold magnification.

### Generation of *Rosa26-Rag* mice for inducible *Rag1/2* activity

In order to generate a mouse line in which Rag1 and Rag2 expression could be induced from the *Rosa26* locus by removal of the STOP cassette upon Cre expression, we used 2A peptides^79^ for bicistronic expression. We amplified the mouse *Rag2* coding region from genomic DNA by PCR reaction with primers (Rag2-5′ and Rag2-2A-3′) which allowed us to add 2A peptide sequences after the stop codon for *Rag2* translation. We also amplified *Rag1* cDNA by PCR. These PCR products were cloned into the pCR-TOPOII vector (Invitrogen), and their sequences verified. We then inserted the *Rag2-2A* fragment in front of the *Rag1* cDNA fragment in pCR-TOPOII, followed by preparation of an entire *Rag2-2A-Rag1* fragment by NotI digestion and a ligation into the NotI site of the pCTV vector (Addgene) (Supp Fig. 2a). The linearized targeting vector was transfected into M1 embryonic stem (ES) cells by electroporation. After G418 selection, ES clones that underwent homologous recombination were screened by PCR as previously described^80^. Primers: Rag2-5′ (5′- CGGCGCGCC AGCATAATTACCAATATGAAAAGATATTC-3′), Rag2-2A-3′ (5′- CGGATCCCCTGGGCCAGGATTCTCCTCGACGTCACCGCATGTTAGCAGACTTCCTCTGCCCTCTCCACTGCCATCAAACAGTCTTCTAAGGAAGGATTTC-3′), Rag1-5′ (5′-GGATCCTATGGCTGCCTCCTTGCCGTCTACCCTGAGC-3′) and Rag1-3′ (5′- CGGCGCGCCATGTGGAGATCCTATTTAAAACTCCATTGA -3′).

### *In vitro* recombination assay

To measure *Rag1/2* activity from the *Rosa26-Rag1A2* locus that was induced upon Cre expression, we used a recombination template vector, pJH200^81^. Embryonic feeder cells generated from E13.5 embryos, which harbour WT or heterozygous *Rosa26-Rag* alleles, were transfected with pJH200 with or without the Cre expression vector, pMC-Cre, using FuGENE reagent (Promega)(Supp Fig. 2b). Three days after transfection, cell lysates were prepared and were analysed for *Rag*-dependent recombination events by PCR, as previously described^81^.

### Stromal culture

Stromal cell culture methods were modified from^58^. In brief, TSt4/DL1 cells (kindly gifted by Dr. Ikawa) were co-cultured with sorted ELCs and DETCs from newborn WT epidermis, in the presence of 10 ng/ml IL-2, 10 ng/ml IL-7, and 10 ng/ml IL-15 (R&D systems), in RPMI medium supplemented with 10% FCS, 2 mM L-glutamine, 1 mM sodium pyruvate, 2 mg/ml sodium bicarbonate, 0,1 mM non-essential amino acids, 50 μM 2-mercaptoethanol, 100 U/ml penicillin, and 100 mg/ml streptomycin.

### Foetal thymic organ cultures (FTOCs)

The experiment was performed as described^82^. 2-deoxyguanosine-treated E15.5 foetal thymic lobes from CD45.1 WT mice were cultured for 7 days before reconstitution with purified ELCs from CD45.2 WT newborns. Around 10 to 15 × 10^3^ ELCs were added to the thymic culture by the Hanging-Drop technique over 24 h, and then cultured for 12–15 days. RPMI medium (Sigma) supplemented with 10% Hyclone FCS (GE Healthcare Life Sciences) 5 μM 2-mercaptoethanol, 10 mM HEPES, 2 mM L-glutamine, 100 U/ml penicillin and 100 μg/streptomycin was used throughout the culture. Medium was additionally supplemented with 1.35 mM of 2’-deoxyguanosine (dGuo) (Sigma) to treat the E15.5 thymic lobes. Treatment with dGuo selectively eliminates thymocytes, interdigitating cells and dendritic cells from the thymic cultured tissue, allowing the introduction of new hematopoietic progenitors to the foetal thymic stromal environment.

### Statistics

Statistical analysis was performed on GraphPad Prism6. Mann-Whitney tests were performed using unpaired experimental designs with non-parametric tests. Significance was defined at p < 0.05 (ns p > 0.05, *p ≤ 0.5, **p ≤ 0.01, ***p ≤ 0.001 and ****p ≤ 0.0001). Error bars in graphs represent Standard Error of the Mean (SEM).

## Additional Information

**How to cite this article**: Almeida, F. F. *et al*. Identification of a novel lymphoid population in the murine epidermis. *Sci. Rep*. **5**, 12554; doi: 10.1038/srep12554 (2015).

## Supplementary Material

Supplementary Information
