# Supplementary material for: Identification of a novel lymphoid population in the murine epidermis
Source: Sci Rep. 2015 Jul 30;5:12554. doi: 10.1038/srep12554 (PMC4519784; doi:10.1038/srep12554)
Supplement: Supplementary Information [file srep12554-s1.pdf]

Identification of a novel lymphoid population in the murine epidermis

Francisca F Almeida[1], Mari Tenno[2], Joanna Brzostek[3], Jackson LiangYao Li[1], Gabriele Allies[4], Guillaume Hoeffel[1], Peter See[1], Lai Guan Ng[1], Hans Jörg Fehling[4], Nicholas R. J. Gascoigne[3], Ichiro Taniuchi[2] and Florent Ginhoux[1]

[1]Singapore Immunology Network (SIgN), Agency for Science, Technology and Research (A*STAR), 138648, Singapore
[2]RIKEN Center for Integrative Medical Sciences (IMS), Japan
[3]Department of Microbiology, Yong Loo Lin School of Medicine, National University of Singapore, Singapore
[4]Institute of Immunology, University Clinics Ulm, Ulm, Germany

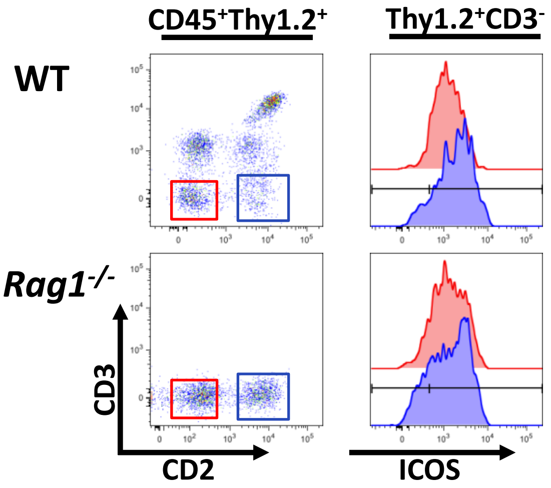

Supp Figure 1.
Thy1^{+} population in the dermis.
Flow cytometry of mouse dermal cell suspension. Gating strategy and histograms to identify ICOS expression across the two populations: Thy1^{+}, CD3^{-}CD2^{-} (red) and CD3^{-}CD2^{+} (blue) in both WT and Rag1$^{-/-}$ mice are shown. Representative data from n>5.

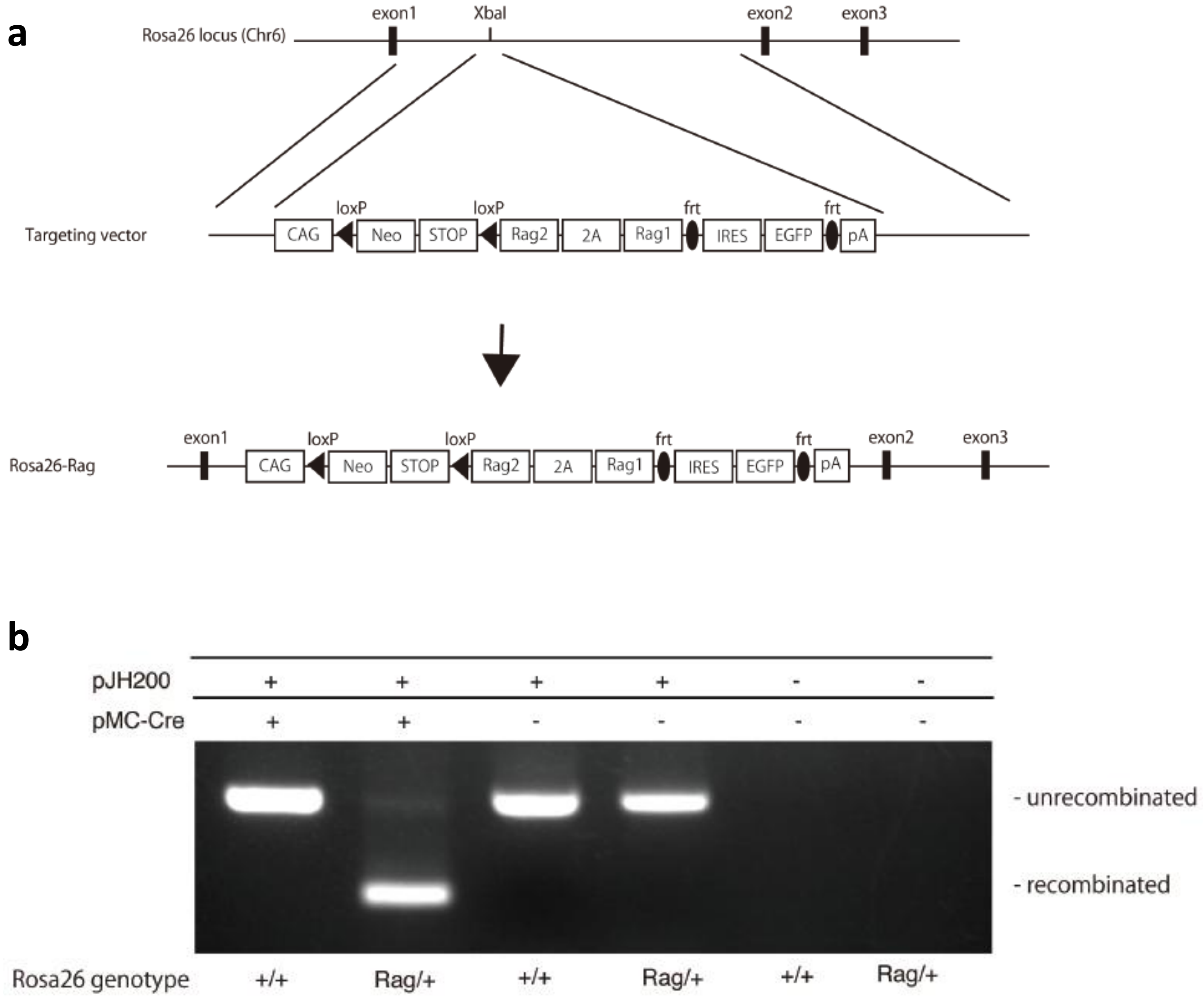

Supp Figure 2.
Generation of *Rosa26-Rag* mouse strain.
a. Strategy for generation of Rosa26-Rag mouse strain by a "knock-in" insertion of *Rag2-2A-Rag1* cDNA fragment into the Rosa26 locus. Structures of the WT *Rosa26* locus, the targeting vector, and *Rosa26-Rag* locus are shown. **b.** Analyses of Rag activity by *in vitro* recombination assay. PCR assay was performed using feeder cells of the indicated genotype, which were transfected with the pJH200 recombination template vector with or without the Cre expression vector, pMC-Cre.

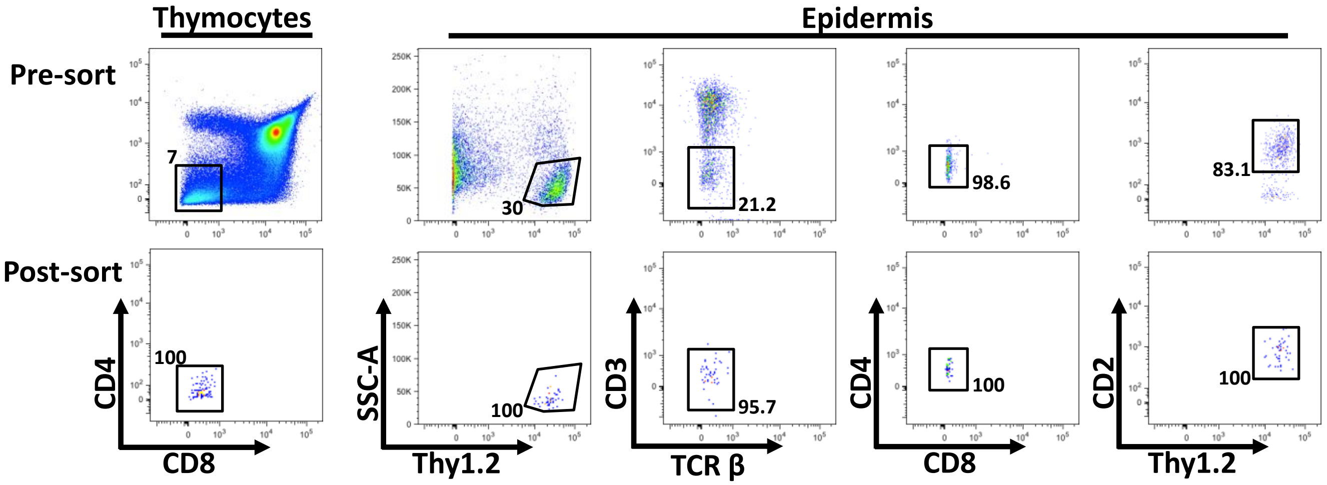

Supp Figure 3.

Sorting strategy and purity of thymic DN cells and ELCs

Gating scheme and post-sort analysis of thymic DN cells (CD4$^-$CD8$^-$) and ELCs from WT NB mice.
